# Exploring mood and anxiety disturbances across Ramadan: a comparative study of Saudi medical students before, during, and after fasting

**DOI:** 10.3389/fpsyg.2025.1570557

**Published:** 2025-06-25

**Authors:** Ayoob Lone, Abdulaziz Shary Hadadi, Ahmed Khalid Alnawah, Alya Abdualrahman Alshammary, Razan Manei Almutairi, Sayed Ibrahim Ali, Naushad Abid

**Affiliations:** ^1^Department of Clinical Neurosciences, College of Medicine, King Faisal University, Alhasa, Saudi Arabia; ^2^College of Medicine, King Faisal University, Alhasa, Saudi Arabia; ^3^Department of Family Medicine, College of Medicine, King Faisal University, Alhasa, Saudi Arabia; ^4^Department of Medicine, College of Medicine, King Faisal University, Alhasa, Saudi Arabia

**Keywords:** Ramadan, intermittent fasting, mood, medical students, Saudi Arabia

## Abstract

Ramadan intermittent fasting (RIF) is a form of diurnal intermittent fasting observed by Muslims worldwide during the month of Ramadan. Previous studies have highlighted various benefits of RIF, particularly in healthy individuals, with positive effects on mood. Despite the physiological demands of RIF, limited research exists on its psychological implications for medical students—a population under considerable academic stress. This study examines changes in the mood of medical students during RIF and explores how these changes may vary according to demographic factors. This prospective study involved 108 medical students who completed the Brunel Mood Scale and demographic questionnaire at three distinct intervals: 1 week before, during, and 1 week after Ramadan. Data was analyzed by repeated measures analysis of variance. The Wilks’ Lambda test was employed to compare the means and evaluate the significant effects of RIF on mood. Statistical significance was set at *p* < 0.05. The findings of this study revealed significant changes in mood during Ramadan. While anger levels remained relatively stable across all three-time points, with only a slight increase during fasting. In contrast, confusion and depression gradually declined, suggesting improved emotional well-being as Ramadan progressed, particularly after fasting ended. Fatigue and tension were highest during the fasting period, indicating greater physical and emotional strain. Meanwhile, vigor temporarily decreased during fasting but returned to higher levels afterward. Result of analysis of variance identified tension as the most significantly affected emotional state (*p* = 0.03). The analysis of emotional states across demographics highlights key significant relationships, with tension showed the strongest associations among the mood dimensions and was significantly affected by academic year (*p* < 0.01) and smoking status (*p* = 0.04). Fatigue showed significant effects across multiple demographics, including area of residence (*p* = 0.03), family structure (*p* = 0.01), sex (*p* = 0.04), income (*p* = 0.02), and smoking status (*p* = 0.01). Confusion was significantly influenced by academic year (*p* = 0.01), while depression showed significant relationships with income (*p* = 0.03) and smoking status (*p* = 0.04). These findings suggest that tension, fatigue, and confusion are particularly sensitive to demographic variations. The study highlights the complex relationship between RIF, emotional outcomes, and sociodemographic variables in medical students, underscoring the need for tailored interventions to support students’ well-being during fasting periods. These findings provide valuable insights into the psychological impact of fasting in the context of medical education.

## Introduction

Ramadan is a ninth month of Islamic calendar. Fasting during Ramadan is a religious practice performed by Muslims. More than 1.8 billion Muslims observe this type of intermittent fasting every year throughout the month of Ramadan. Islamic law states that while all Muslims are encouraged to observe the Ramadan fast for 29–30 days (Mosaferchi et al., 2020). Individuals who are unwell, young children, elderly, pregnant, nursing, menstruating, or traveling are exempted from fasting (Alghafli et al., 2014). The length of the daily fast during Ramadan varies based on the season and region, ranging from 12 to 18 h (Bandarian et al., 2021). People who observe fasting are entirely refrain from eating or drinking from dawn until sunset. Most Muslims have two meals during Ramadan: the sahur, which is eaten shortly before dawn, and the iftar, which is eaten shortly after sunset. During Ramadan, people refrain from eating and drinking, as well as from restraining their entire body, including the tongue, eyes, and ears, from undesirable behaviors. Thus fasting improves self-control and self-discipline among Muslims. Consequently, it is believed that the physiological changes that occur during Islamic fasting are distinct from those that occur during experimental fasting (Kartal et al., 2021; Ghazi et al., 2018). While fasting primarily holds religious significance, there has been growing interest in exploring its psychological and physiological effects, especially in specific populations such as students. In particular, medical students, who are often under intense academic and emotional pressure due to the demanding nature of their training, provide a unique population for examining the relationship between fasting and mood.

Mood is commonly defined in the psychological literature as a prolonged emotional state that is less intense than emotions but more persistent and pervasive. Unlike emotions, which are typically triggered by specific events, moods can arise without clear external causes and tend to influence an individual’s overall emotional outlook over a longer period (Beedie et al., 2005; Siemer, 2009). Moods are generally considered to be lower in intensity compared to emotions, but they can last for hours, days, or even longer, affecting cognition, behavior, and perception during that time. Understanding the distinction between the two is important, especially when investigating how fasting during Ramadan might influence emotional well-being, as both mood and emotions may be affected in different ways.

Medical students are known to experience significant emotional strain, often manifesting as stress, anxiety, and depression, largely due to the intense workload and high expectations associated with their training (Dyrbye et al., 2005; Yusoff et al., 2013; Melaku et al., 2021). For these students, RIF may introduce additional physical and emotional challenges—such as disruptions in sleep patterns, changes in daily routines, and the psychological impact of fasting itself—while also being associated with potential psychological benefits, including improved self-control, enhanced focus, and a sense of spiritual fulfillment (Alnashri et al., 2022; Faris et al., 2020; Lansang and McDonald, 1998). The emotional impact of fasting in the context of medical education remains understudied.

There are various benefits of fasting including physical, emotional, social and mental well-being (Yousuf et al., 2021; Nugraha et al., 2017). Previous research has explored the general effects of fasting on emotional well-being, noting both positive and negative outcomes. These outcomes are often shaped by factors such as the type of fasting (e.g., intermittent fasting, religious fasting), the duration of the fast, the season during which the Ramadan is conducted, the climate zone, individual psychological resilience, and the context in which fasting takes place (e.g., religious, health-related, or for weight loss). Some research suggests that fasting may improve mood, leading to higher levels of positive emotions and vitality, and also reducing negative emotions (Watkins and Serpell, 2016; Moreno-Domínguez et al., 2012; Erdem, 2018; Hussin et al., 2013; Wang and Wu, 2022; Berthelot et al., 2021; Akan et al., 2023). While others findings have indicated that fasting could lead to irritability, anxiety, anger, fatigue, or mood disturbances (Appleton and Baker, 2015; Bolton et al., 2014; Solianik et al., 2016). These findings point to the complexity of fasting’s emotional impact, which may be modulated by factors such as the individual’s pre-existing emotional state, coping strategies, and the specific environment in which fasting takes place.

Despite the increasing attention on the psychological and physiological effects of RIF, few studies have specifically focused on how fasting may influence the mood of medical students, who face a complex combination of stressors and demands. Studying this group provides critical insight into how fasting interacts with pre-existing psychological pressures in a high-performing population. Understanding how fasting affects mood and emotional regulation in this population is critical for developing targeted interventions that can help students maintain their well-being during Ramadan. This study examines changes in the mood of medical students during RIF and explores how these changes may vary according to demographic factors. This research seeks to provide valuable insights into the psychological effects of fasting within the specific context of medical education. Based on this rationale, we hypothesize that medical students will exhibit significant mood fluctuations across the three phases of Ramadan (before, during, and after fasting). Specifically, we anticipate increased negative mood states—such as fatigue and tension—during fasting, followed by improvements in mood, including reduced depression and confusion, after Ramadan. We further expect that these mood changes will differ based on demographic variables such as sex, academic year, and smoking status.

## Materials and methods

### Study design

This prospective study was conducted between March and May, 2024 on undergraduate medical students. This research granted ethical approval from the Deanship of Scientific Research at King Faisal University, Alhasa, Saudi Arabia. This research was conducted in accordance with the Helsinki Declaration on research involving human participants. One month before Ramadan, we contacted a student’s representative of medical college as each batch of students in the college has its own social network group through which they shares information. We contacted the administrator of the social network (WhatsApp) group to inform the batch-mates about the study, and their email addresses were then obtained. Prior to participation, all study subjects were informed about the purpose and goal of the research, and informal consent was obtained in three stages between 1 week before Ramadan, during 2nd and 3rd week of Ramadan and 1 week after Ramadan were given. This study adopted a three-stage data collection design—administering the Brunel Mood Scale (BRUMS) before, during, and after Ramadan—to comprehensively capture temporal mood fluctuations associated with the fasting period. This approach allows for the assessment of emotional states at distinct phases of the fasting cycle: (1) the anticipatory period prior to fasting, (2) the physiological and psychological impact during fasting, and (3) post-fasting emotional adjustments. Such a design provides a more nuanced understanding of how mood states evolve across the Ramadan experience.

### Participants

The participants of the present study were recruited from the college of medicine, King Faisal University, Alhasa Saudi Arabia. A stratified sampling method was applied to ensure that the sample was representative of the population. The representative sample was first divided into different groups based on their academic year, socioeconomic status, and family structure ensuring each group was proportionality representative in the final sample. A random sample was then applied from each of these strata to ensure comprehensive representation. A total of 154 medical students were recruited to participate in this study voluntarily. In the first stage, 135 (out of 154) participants fill the survey. During the second stage, the response rate was reduced to 115 out of 154 participants. Out of one hundred fifty four sample, 108 participants completed all stages of the survey with a 70.13% response rate. Missing data from 46 (29.87%) participants were not included in the final analysis. The low response rate was primarily due to the fact that majority of students were already taking their semester examinations. The sample size for this study was calculated using Slovin’s formula, based on a total population of 220 participants, as reported in a previously published study by Alotaibi et al. (2023) with a confidence interval of 0.95 and margin of error of 0.05.

### Inclusion and exclusion criteria

Participants were deemed eligible for inclusion if they were medical students aged between 19 and 25 years, were physically and mentally capable of fasting, and had observed RIF in the past. Exclusion criteria included non-medicals student, had chronic diseases, and receiving medical treatment, past surgery, pregnant and lactating women and history of psychological problem.

### Measures

In order to achieve the aim of the present study, we used Brunel Mood Scale for measuring mood of the study participants. This study also included demographic questionnaire prepared by the researchers.

### Brunel Mood Scale

A previously validated self-reported questionnaire was used to assess the mood of the participants. This scale was developed originally for use with adolescents and athletes and it has been validated for use with a wide range of population (Terry et al., 1999). This 24-item scale comprised of six subscales of four item each, (*1. T*ension: anxious, nervous, panicky, worried, *2*. Depression: downhearted, miserable, unhappy, depressed, *3.* Anger: annoyed, angry, bitter, energetic, *4.* Vigor: actively, energetic, lively, alert, *5.* Fatigue: exhausted, tired, worn out, sleepy, *6.* Confusion: mixed up, muddled, uncertain, confused). A 5-point Likert scale with 0 representing “not at all” and 4 “extremely” was used by respondents to rate their mood, with total possible subscale scores ranging from 0 to 16. In order to obtain overall mood score referred to as total mood disturbances score, can be calculated by summing the score for subscale such as tension, depression, anger, fatigue and confusion and then subtracting the vigor score. Previous research has reported internal consistency reliability (Cronbach alpha coefficients) for Brunel Mood Scale ranging from 0.74 to 0.90 (Terry et al., 2003). For the present study, Cronbach alpha coefficients was ranging from 0.71 to 0.89.

### Demographic questionnaire

This questionnaire covered questions related to demographic information such as sex, age, and academic year. In addition, details about their families such as, areas of residence, family type, and socio-economic status. Furthermore, some questions relate to their lifestyle: consumption of caffeine and smoking status were also asked.

### Procedure

After receiving the required approval from the college authorities, the participants were approached personally by the trained medical students. Participants were briefed on the purpose of the study and the questionnaire that would be used for data collection. Upon obtaining their consent, a suitable time and date for the data collection were arranged. Before the questionnaire was administered, the study’s objectives were once again explained to the participants, and they were assured that their responses would be kept confidential and used solely for academic and research purposes. A rapport was built with the participants to ensure they felt comfortable providing honest and accurate responses. Participants were given clear instructions and guidance on how to complete the questionnaire correctly. The questionnaires were then distributed, and participants were asked to complete them according to the provided instructions. It took approximately 10 min to finish the questionnaire. Afterward, the completed questionnaires were collected, and the participants were thanked for their time and cooperation.

### Statistical analysis

The responses from the Brunel Mood Scale and demographic data were summarized using means and standard deviations. Repeated measures ANOVA was conducted to analyze mood changes across three periods (before, during, and after Ramadan). Wilks Lambda test was applied to compare means and assess the impact of RIF on mood. Statistical significance was set at *p* < 0.05. Effect sizes were calculated using eta squared (η^2^), interpreted according to Cohen’s thresholds: small (η^2^ = 0.01), moderate (η^2^ = 0.06), and large (η^2^ = 0.14). Mauchly’s Test was used to evaluate the assumption of sphericity. For variables violating this assumption, degrees of freedom were adjusted using Greenhouse–Geisser estimates to ensure valid interpretations. All statistical analyses were performed using IBM SPSS Statistics (Version 27.0, Chicago, IL, United States) (IBM Corp, 2020).

## Results

Table 1 provides a detailed breakdown of the demographic characteristics of the study sample. The sex distribution reveals a predominance of female participants (64.8%) over male participants (35.2%), which may affect the generalizability of the findings, especially in terms of sex-specific emotional responses.

**Table 1:** General characteristics of the participants in the study (*N* = 108).

Characteristics		*n*	%
Sex	Male	38	35.2%
	Female	70	64.8%
Academic year	1st year	13	12.0%
	2nd year	10	9.3%
	3rd year	22	20.4%
	4th year	57	52.8%
	5th year	6	5.6%
Family status	Joint	97	89.8%
	Nuclear	11	10.2%
Monthly income	$1,333	15	13.9%
	$1,333–$2,666	13	12.0%
	$2,667–$4,000	15	13.9%
	>$4,000	65	60.2%
Number of family members	≤3	9	8.3%
	4	4	3.7%
	5	10	9.3%
	6	19	17.6%
	7	23	21.3%
	8	16	14.8%
	9	17	15.7%
	≥10	10	9.3%
Smoking status	Non-smoker	104	96.3%
	Smoker	4	3.7%
Area of residence	Rural	24	22.2%
	Urban	84	77.8%
Age in years (mean ± SD)		22.26 ± 0.95	

The sample is heavily skewed towards advanced academic years, with 52.8% in their 4th year, and a relatively small representation from the first year (12.0%) and 5th year students (5.6%). This concentration in later academic years may influence the study’s outcomes related to academic stress. Family status shows a strong preference for joint family systems (89.8%) over nuclear families (10.2%), reflecting potential cultural influences. Additionally, a significant majority of participants report an income greater than >$4,000 (60.2%), which could impact the study’s findings on income-related variables. Furthermore, the data revealed that most participants come from larger families, with 37.0% having seven or more members. The sample is predominantly non-smokers (96.3%), with only a small percentage of smokers (3.7%), and mostly urban residents (77.8%). The mean age of the sample is 22.26 years, reflecting the typical age range for undergraduate students.

Table 2 presents the mean and standard deviation for six emotional states—anger, confusion, depression, fatigue, tension, and vigor—measured before, during, and after fasting in Ramadan. The results show that anger levels remained relatively stable across the three time points, with a slight increase during fasting (M = 6.89, SD = 3.06) compared to before (M = 6.63, SD = 3.19) and after fasting (M = 6.45, SD = 3.18). In contrast, confusion and depression levels decreased over time, with confusion dropping from a mean of 7.36 before fasting to 6.10 after fasting, and depression showing a similar downward trend. This suggests that participants experienced less confusion and depression as Ramadan progressed, particularly after the fasting period ended.

**Table 2:** Means, standard deviations of Brunel Mood Scale (*n* = 108).

Brunel Mood Scale	Before Ramadan		During Ramadan		After Ramadan	
	Mean	SD	Mean	SD	Mean	SD
Anger	6.63	3.19	6.89	3.06	6.45	3.18
Confusion	7.36	3.55	6.70	3.53	6.10	3.86
Depression	6.38	4.29	6.25	4.67	5.66	4.23
Fatigue	8.88	3.63	9.99	3.78	8.33	3.74
Tension	6.92	4.01	8.31	4.20	7.76	3.79
Vigor	7.81	3.55	7.24	2.98	7.97	3.92

**Figure 1:**
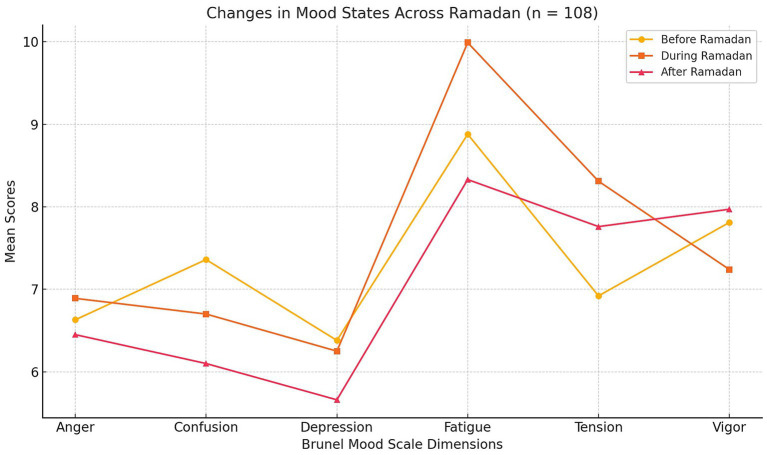
Mean Brunel Mood Scale scores before, during, and after Ramadan among medical students (*n* = 108), showing trends across six mood dimensions.

Fatigue and tension, however, were highest during fasting, with fatigue increasing from a mean of 8.88 before fasting to 9.99 during fasting, and tension rising from 6.92 to 8.31 in the same period. These results indicate that participants experienced more physical and emotional strain during fasting. Conversely, vigor showed a temporary decline during fasting, dropping from 7.81 before fasting to 7.24 during fasting, but then rebounded after fasting to 7.97. Overall, these findings suggest that while some emotional states, such as confusion and depression, improved after fasting, others, like fatigue and tension, peaked during the fasting period, reflecting the challenges associated with fasting in Ramadan. The mean scores for the six mood dimensions across the three phases of Ramadan (before, during, and after) are graphically presented in Figure 1. This figure illustrates the variations in Brunel Mood Scale scores over time among the participating medical students.

Mauchly’s Test assesses the assumption of sphericity, a key requirement for repeated measures analysis of variance. Result presented in Table 3 indicated that most variables of Brunel Mood Scale including anger, confusion, depression, fatigue, and vigor were not significant (*p* > 0.05), indicating that the assumption of sphericity is met. This implies that no corrections to the degrees of freedom are necessary for these variables. However, tension shows a significant result (χ2 = 8.07, *p* = 0.02), suggesting a violation of sphericity. To address this, adjusted epsilon values (Greenhouse–Geisser = 0.93, Huynh-Feldt = 0.94, and Lower-bound = 0.50) should be used to correct the degrees of freedom when interpreting the analysis of variance results for tension. These adjustments ensure the validity of the statistical tests while accounting for the violation of sphericity.

**Table 3:** Mauchly’s test of sphericity for emotional states measured by the Brunel Mood Scale.

Within subjects effect	Mauchly’s W	Approx. Chi-Square	df	*P* value	Epsilon		
					Greenhouse–Geisser	Huynh-Feldt	Lower-bound
Anger	0.99	0.93	2	0.62	0.99	1.00	0.50
Confusion	0.97	3.06	2	0.21	0.97	0.99	0.50
Depression	0.97	2.68	2	0.26	0.97	0.99	0.50
Fatigue	0.99	0.33	2	0.84	0.99	1.00	0.50
Tension	0.92	8.07	2	0.02*	0.93	0.94	0.50
Vigor	0.97	2.36	2	0.30	0.97	0.99	0.50

* *p* < 0.05.

The results of analysis of variance presented in Table 4 revealed that among the six emotional states analyzed, only tension demonstrated a statistically significant effect (*F* (2,106) = 4.54, *p* = 0.03), indicating that the independent variable significantly influenced this emotion. The effect size, as shown by Partial Eta Squared (0.08), suggests a small-to-moderate impact. This highlights tension as the primary emotion impacted, warranting further investigation into its contributing factors or experimental conditions. The remaining emotional states—anger, confusion, depressed, fatigue, and vigor—did not show statistically significant effects (*p* > 0.05).

**Table 4:** Impact of Ramadan intermittent fasting on mood using repeated measures analysis of variance.

Source	Type III	df	Mean Square	*F*	*P* value	Partial Eta Squared
	Sum of Squares					
Anger	21.98	2	10.99	2.20	0.15	0.22
Confusion	0.17	2	0.08	0.01	0.99	0.01
Depressed	8.76	2	4.38	0.83	0.45	0.03
Fatigue	26.31	2	13.15	1.43	0.27	0.11
Tension	14.09	2	7.04	4.54	0.03*	0.08
Vigor	9.61	2	4.80	1.83	0.20	0.13

* *p* < 0.05.

However, anger and vigor demonstrated moderate effect sizes (0.22 and 0.13 respectively), hinting at potential trends that may become significant with a larger sample size or refined methodology. Conversely, confusion exhibited negligible effects (η2 = 0.01), suggesting no meaningful impact. These findings suggest that while most emotional states remain unaffected, certain trends merit closer scrutiny in future studies.

The analysis of emotional states across demographics presented in Table 5 highlights key significant relationships. Tension aspect of Brunel Mood Scale exhibited the strongest associations, significantly affected by academic year (*F* = 3.65, *p* < 0.01) and smoking status (*F* = 2.27, *p* = 0.04). Additionally, fatigue showed significant effects across multiple demographics, including area of residence (*F* = 2.53, *p* = 0.03), family structure (*F* = 3.26, *p* = 0.01), sex (*F* = 2.33, *p* = 0.04), income (*F* = 2.05, *p* = 0.02), and smoking status (*F* = 3.40, *p* = 0.01). Confusion was significantly influenced by academic year (*F* = 2.21, *p* = 0.01), while depression showed significant relationships with income (*F* = 1.98, *p* = 0.03) and smoking status (*F* = 2.38, *p* = 0.04). These findings suggest that tension, fatigue, and confusion are particularly sensitive to demographic variations.

**Table 5:** Repeated-measure analysis of variance for emotional states with demographic variables.

Brunel Mood Scale	Demographic factors	F-statistic	*P* value
Anger	Academic year	1.34	0.18
Anger	Area of residence	0.28	0.92
Anger	Family status	1.54	0.17
Anger	Sex	0.64	0.66
Anger	Monthly income	1.26	0.24
Anger	Smoking status	0.48	0.79
Confusion	Academic year	2.21	0.01**
Confusion	Area of residence	1.77	0.11
Confusion	Family status	1.94	0.08
Confusion	Sex	2.00	0.07
Confusion	Monthly income	1.36	0.18
Confusion	Smoking status	1.71	0.13
Depression	Academic year	1.44	0.13
Depression	Area of residence	1.10	0.35
Depression	Family status	0.66	0.65
Depression	Sex	2.14	0.06
Depression	Monthly income	1.98	0.03*
Depression	Smoking status	2.38	0.04*
Fatigue	Academic year	1.62	0.19
Fatigue	Area of residence	2.53	0.03*
Fatigue	Family status	3.26	0.01**
Fatigue	Sex	2.33	0.04*
Fatigue	Monthly income	2.05	0.02*
Fatigue	Smoking status	3.40	0.01**
Tension	Academic year	3.65	0.01**
Tension	Area of residence	1.66	0.14
Tension	Family status	1.64	0.14
Tension	Sex	1.70	0.13
Tension	Monthly income	1.64	0.08
Tension	Smoking status	2.27	0.04*
Vigor	Academic year	1.56	0.08
Vigor	Area of residence	0.79	0.55
Vigor	Family status	0.64	0.66
Vigor	Sex	0.71	0.61
Vigor	Monthly income	0.85	0.58
Vigor	Smoking status	1.08	0.37

* *p* < 0.05, ** *p* < 0.01.

In contrast, anger and vigor demonstrated no significant associations with any of the demographic variables, indicating a more uniform distribution across these groups. The absence of significant effects for anger suggests minimal demographic influence, while vigor’s lack of significant relationships aligns with its nature as a positive emotion less affected by external factors. These insights underscore the importance of considering demographic variables in understanding emotional responses, particularly for tension, fatigue, and confusion, which show clear susceptibility to contextual and personal factors.

## Discussion

To the best of our knowledge, this is the first study conducted in Saudi Arabia that explores changes in various dimensions of the Brunel Mood Scale during RIF and examines how these changes are associated with demographic variables. The findings of this study revealed that RIF has significant influence on all dimensions of mood scale. The result shows that anger levels remained relatively stable across the three time points, with a slight increase during fasting compared to before and after fasting. Previous studies has reported that emotional shifts such as heightened anger and aggression were noted in a group of amateur weightlifters during 48 h fasting, while there were no significant changes in mental flexibility or task-switching abilities (Solianik et al., 2016). The slight increase in anger during fasting, with relative stability before and after, aligns with physiological and psychological models explaining the body’s emotional response to hunger and fasting (Solianik et al., 2016; Wang and Wu, 2022). Fasting induces physiological stress due to lower glucose levels, which can impair emotion regulation and lead to heightened irritability or aggression. However, as individuals adapt to the fasting state, their emotional responses stabilize. The return to baseline anger levels before and after fasting reflects this adaptation and the transient nature of fasting-induced emotional fluctuations.

The findings of the study revealed that participants experienced less confusion and depression as Ramadan progressed, particularly after the fasting period ended. This phenomenon can be attributed to several factors, including adaptation to fasting, changes in hormonal balance, the psychological benefits of completing a challenging but spiritually fulfilling practice like Ramadan, and physical recovery following the fasting period. As the body adjusts to the fasting regimen, individuals often report better cognitive function, improved mood, and a sense of well-being. Additionally, the physical relief and improved social connections at the end of Ramadan may further contribute to the observed decrease in confusion and depression. Importantly, the spiritual nature of Ramadan—marked by increased prayer, reflection, and mindfulness—also plays a critical role in enhancing psychological stability. The sense of inner peace and connection with faith that many individuals experience during this month may help buffer emotional distress and foster resilience, contributing to the reduction in negative emotional states such as confusion and depression (Jandali et al., 2024; Aldbyani, 2025). These findings align with earlier research that has documented the psychological benefits of Ramadan fasting in terms of improved emotional regulation, reduced stress, and enhanced well-being. Specifically, studies have linked these outcomes to both behavioral and biological mechanisms, including reductions in inflammatory markers (Faris et al., 2020; Faris et al., 2019; Liu et al., 2024) and favorable gene expression changes related to neuroplasticity and inflammation (Madkour et al., 2022; Madkour et al., 2019). Such physiological adaptations during RIF may contribute significantly to the observed improvements in mood and mental health among fasting individuals. In addition to spiritual, behavioral, and immunological mechanisms, the improvement in mental health observed during RIF may also be underpinned by neurobiological factors, particularly the modulation of brain-derived neurotrophic factor (BDNF). BDNF is essential for synaptic plasticity, cognitive function, and emotional regulation. Dysregulation of BDNF has been implicated in depression and anxiety disorders. Emerging evidence indicates that intermittent fasting, including RIF, can lead to a physiological upregulation of BDNF, which contributes to enhanced neuronal resilience and psychological stability (Alkurd et al., 2024).

These results indicate that participants experienced more fatigue and tension during fasting compared to pre- and post-fasting periods. This aligns with existing literature suggesting that all forms of fasting can lead to increased fatigue (Zajac et al., 2021; Alkandari et al., 2012). During fasting, the body undergoes significant hormonal, metabolic, inflammatory, neuroendocrine, and circadian changes (Al-Rawi et al., 2020; Faris et al., 2021; Tinsley and La Bounty, 2015), including increases in cortisol, adrenaline, and other stress-related hormones. Cortisol, commonly referred to as the “stress hormone,” is released by the hypothalamic–pituitary–adrenal (HPA) axis to help the body adapt to periods of energy scarcity, including fasting. However, chronic elevation or disruption in cortisol secretion can impair sleep quality, elevate psychological stress, and contribute to fatigue and emotional instability. While many forms of fasting can lead to increased fatigue due to a combination of metabolic shifts, dehydration, hormonal changes, and disrupted circadian rhythms, RIF presents a unique hormonal profile due to its diurnal and dry nature. In contrast to other forms of intermittent fasting, RIF is associated with a delay and flattening in the normal diurnal rhythm of cortisol secretion. Previous researches reported that individuals observing RIF show a postponed peak in cortisol levels and altered morning-to-evening cortisol ratios, likely due to changes in meal timing, sleep–wake cycles, and physical activity during Ramadan (Al-Rawi et al., 2020). These hormonal adaptations may reflect the body’s attempt to recalibrate its internal stress response to the fasting schedule. However, this shift in cortisol dynamics may also contribute to the feelings of fatigue and tension often reported during the fasting period, especially in the early days of Ramadan. Thus, the increase in fatigue observed among participants in our study may be partially explained by this altered HPA axis activity, compounded by sleep disturbances, reduced caloric intake, and psychosocial stressors. However, in Islamic tradition, fasting is not only a physical act of abstention but also a spiritual exercise in self-control and moral refinement.

Additionally, vigor showed a temporary decline during fasting, which was better before and after the fasting. Our results are consistent with the previous findings (Kaarud et al., 2016), which suggested that vigor was decreases while fatigue increases in Ramadan during exercise among university students. The decline in vigor during fasting, with improvement before and after, is primarily due to the body’s energy depletion during the fasting period. As glucose levels drop and the body adjusts to fasting, energy levels temporarily decrease, leading to reduced vigor. Once normal eating resumes, energy stores are replenished, and vigor returns to baseline or even improves. Moreover, fasting can result in lower energy availability, particularly during extended periods without food. This depletion affects muscle function, stamina, and overall vitality. During fasting, the body prioritizes essential functions, and as a result, physical and mental vigor may temporarily decrease. Once the fasting period ends and regular eating resumes, the body replenishes energy stores (such as glycogen), leading to a rebound in vigor levels.

Interestingly, RIF is associated with improvements in mental health status. The results indicated that tension was heightened when individual observed fasting. The increased tension experienced during fasting, compared to pre- and post-fasting periods, is a result of a combination of hormonal changes (e.g., elevated cortisol), physiological responses (e.g., reduced glucose levels and dehydration), and psychological factors (e.g., hunger, food deprivation, and sleep disturbances) (Wang and Wu, 2022). These factors contribute to heightened stress and tension, which tend to be most pronounced during the fasting period itself, when the body is adjusting to the lack of food and undergoing significant metabolic and hormonal shifts. During fasting, blood glucose levels drop, leading to a reduction in available energy for the brain and muscles. The brain, which is highly dependent on glucose for optimal function, becomes stressed when its energy supply is limited. This can result in feelings of irritability, poor concentration, and tension, particularly in the early stages of fasting when the body is transitioning to using fat and ketones for energy (Fairclough and Houston, 2004; Scholey et al., 2001). Research consistently supports these mechanisms, showing that fasting can lead to significant psychological and physiological stress, manifesting as increased tension (Wang and Wu, 2022).

Additionally, we examined the relationship of demographic variables and RIF. Results clearly revealed that demographic characteristics significantly affects different aspects of mood such as, confusion, depression, fatigue and tension. Based on the analysis of significant differences, the results show that first-year students experienced higher levels of confusion during fasting as compared to other academic years. Presently there is no similar studies for comparison. However, previous studies shows that medical students in their basic science years reported high rates of mental health problems such as anxiety, depression, and imposter syndrome (Mazurkiewicz et al., 2012; Thompson et al., 2016; Boni et al., 2018; Salana et al., 2020; March-Amengual et al., 2022). The observation that first-year students experienced higher levels of confusion during fasting compared to other academic years can be logically explained by several factors: First-year students are often required to learn new material at a faster pace, which may lead to higher cognitive load. When combined with the physiological effects of fasting (such as low blood sugar levels), this increased cognitive load could result in difficulty processing and recalling information, leading to confusion. Moreover, the stress of managing coursework, assignments, exams, and adapting to a new academic environment may be more pronounced for first-year students. Fasting can amplify stress and anxiety, which may impair mental clarity and contribute to confusion. First-year students might not yet have developed the coping mechanisms that upper-year students have refined over time.

For depression aspect of Brunel Mood Scale, participants with income between $1,333–$2,666 experience higher level of depression due to Ramadan intermittent fasting. There is not relevant research for comparison, however, few studies reported that rates of depression anxiety and suicide correlated negatively with income (Sareen et al., 2011; Lund et al., 2010; Iemmi et al., 2016; Banks et al., 2017). Those with the lowest income suffer more frequently from depression, anxiety and other common mental illness than those with the highest income (Sareen et al., 2011; Frasquilho et al., 2016; Madigan and Daly, 2023). People within the income range of $1,333–$2,666 might experience more financial stress during Ramadan. Even though this income is considered middle-income in some regions, the added pressure of increased expenses (e.g., food, gifts, family gatherings) during Ramadan could cause financial strain. Additionally, the rising costs of living upon the observance of Ramadan, especially if there are additional family responsibilities or unplanned expenditures, could lead to feelings of stress and depression. Furthermore, Ramadan is a time of community, charity, and family gatherings. People within this income group may feel pressure to participate in social events, give to charity, or host family gatherings, which can lead to emotional and financial strain. There might also be an expectation to provide a certain standard of living during this time (luxurious iftar meals, family entertainment), which can create stress for individuals in this income bracket who may not have the resources to meet these expectations. Results of this study also indicated that smokers reported significantly higher levels of depression during Ramadan. Our results are partially in accordance with previous studies (Kadri et al., 2000), which reported that irritability was significantly higher in smokers than non-smokers before the beginning of Ramadan and increases in both groups during Ramadan and its peak at the end at the end of the month. For many smokers, cigarettes are a coping mechanism for stress, anxiety, or negative emotions. Without access to cigarettes during fasting hours, smokers may struggle with managing their emotions, leading to a sense of heightened stress, frustration, and sadness. Moreover, during Ramadan, smokers are more likely to become acutely aware of their cravings, especially when they face stressful situations. This heightened awareness can amplify feelings of anxiety and irritability, contributing to an increased likelihood of depressive symptoms.

Regarding fatigue, the analysis revealed that participants living in rural areas, having joint family system, being male, low monthly income and smokers were experienced more fatigue during Ramadan. Fatigue is the state of feeling very tired, resulting from insufficient sleep, prolonged mental and physical tiredness and apathy (Valko et al., 2008). Research on RIF and fatigue often shows that sleep deprivation, dehydration, and poor nutrition are common factors contributing to fatigue during the month of Ramadan (Bouhlel and Shephard, 2015; BaHammam and Almeneessier, 2020; Chtourou et al., 2011; Singh et al., 2011). Individuals in rural areas are engaged in physically demanding work, such as farming, construction, or other labor-intensive jobs. Fasting during Ramadan while performing strenuous physical activities can lead to greater fatigue compared to individuals in urban areas who might have access to more sedentary or controlled environments. Result shows that participants from joint family experienced more fatigue as compare to the participants from nuclear families. To the best of our knowledge, similar studies have not been carried out in people living in joint families, which makes it difficult to compare our results. The joint family system is a family structure in which extended families, including grandparents, parents, children, uncles, aunts, and cousins, all live together in one household. The joint family system can increase fatigue during Ramadan due to a combination of physical strain, emotional labor, disrupted sleep, and social pressures. Managing household tasks for a larger group, caregiving duties, and balancing social expectations can be overwhelming, particularly for women who often shoulder the brunt of household work. It is obvious that additional responsibilities, combined with the challenges of fasting, can lead to higher levels of fatigue, especially in a joint family structure. Regarding sex, the results shows significant difference with respect to the fatigue, specifically, male students experienced more fatigue during Ramadan as compare to female students. Men generally have a higher basal metabolic rate than women (Jagim et al., 2023). This means that they burn more calories even at rest, which could lead to greater energy consumption throughout the day. During Ramadan, fasting may strain their energy levels more significantly than for women, leading to greater fatigue. Moreover, men typically have more muscle mass than women, and muscles require more energy to function. During the long hours of fasting, this increased energy demand can lead to greater physical fatigue in men compared to women.

The other important new finding is that participants with low income experience more fatigue than those having high monthly income. Previous studies reported that low income is demonstrably correlated with poor mental health, but it is less certain whether increasing a person’s income will lead to improvements in their mental well-being (Reiss, 2013; Mackenbach, 2020). During Ramadan, those with lower incomes may experience heightened financial stress due to the increased costs of food and other necessities for Suhoor (pre-dawn meal) and Iftar (meal to break the fast). The added financial burden can lead to mental fatigue, making it harder for them to manage the physical challenges of fasting. Moreover, lower-income individuals might feel more pressure to provide for their families, especially during Ramadan, when social and religious obligations may increase. This added stress can contribute to both mental and physical fatigue. Furthermore, individuals with low incomes are more likely to experience mental health challenges, such as anxiety, depression, and stress (Frasquilho et al., 2016; Madigan and Daly, 2023). These conditions can worsen during Ramadan, especially when coupled with the pressures of fasting and financial difficulties. Mental health issues can directly contribute to feelings of fatigue and exhaustion, making it harder to cope with the physical demands of fasting. Another significant factor was found in the present study, participants with smoking history reported more fatigue during Ramadan as compared to non-smokers. Our results are partially compatible with those in the literature. These researches suggested that Muslim smokers experienced lower self-esteem (Caliyurt et al., 2005), and greater irritability during RIF (Kadri et al., 2000). The result that participants with a smoking history reported more fatigue during Ramadan compared to non-smokers can be logically explained through several physiological, behavioral, and health-related factors. Smokers are often dependent on nicotine, which is a stimulant that affects the central nervous system (Singh et al., 2023). During Ramadan, smokers refrain from smoking for long hours due to fasting. The absence of nicotine can lead to withdrawal symptoms, including irritability, restlessness, anxiety, increased hunger and eating, insomnia, difficulty concentrating, and fatigue (Hughes and Hatsukami, 1986). Nicotine withdrawal can cause physical and mental exhaustion, making the fasting period more challenging.

Interestingly, our results suggest that certain demographic such as, academic years and smoking status were associated with higher levels of tension during Ramadan intermittent fasting. The analysis of significant differences reveals that first-year medical students reported higher levels of tension during Ramadan compared with students in other academic years. Currently, there is no similar study for comparison, however, Guthrie et al. (1998) reported that the students found medical course stressful during the first year of study but less so in subsequent years. First-year medical students likely report higher levels of tension during Ramadan due to a combination of factors, including the academic pressures of adjusting to a challenging medical curriculum, inexperience with fasting while managing schoolwork, increased social and cultural expectations, physical strain from fasting, and emotional sensitivity. These factors make the transition to medical school particularly stressful during Ramadan, contributing to elevated tension levels compared to students in later academic years. Multiple studies have demonstrated an association between cigarette smoking and mental health problems (Moylan et al., 2013; Dome et al., 2010; Wu et al., 2023; Fluharty et al., 2017). Our results shows that smokers experienced more tension upon the observance of Ramadan compared to non-smokers. Smokers are physically dependent on nicotine, a stimulant that has mood-regulating effects. During Ramadan, smokers refrain from smoking for extended periods, leading to withdrawal symptoms such as irritability, anxiety, and increased tension (Hughes and Hatsukami, 1986). These symptoms are especially pronounced in habitual smokers who are used to frequent nicotine intake, making the fasting experience more stressful. Moreover, many smokers use cigarettes as a way to cope with stress, anxiety, or difficult emotions. Without this coping mechanism during Ramadan, smokers may find it harder to manage feelings of tension and stress. The absence of nicotine, combined with the demands of fasting, can create a sense of unease and exacerbate pre-existing stress levels.

When analyzing the results of this study, it is important to take into account several limitations. One limitation of this study concerns its small sample size, so the results should be interpreted with caution. This study is susceptible to selection bias, as participants were recruited from medical colleges in the Eastern Governorate of Saudi Arabia. As a result, the generalizability of the findings should be carefully considered. The dropout rate (29.87%) may have been influenced by fasting-related fatigue, academic burden, or survey fatigue, which could also introduce response bias.” Additionally, this study focused solely on the fasting group. Therefore, the absence of a non-fasting control group restricts the ability to isolate fasting-related effects. Future research should include control groups for comparative analysis. Due to the underrepresentation of male participants in our sample, we were unable to draw strong conclusions about sex differences in mood responses. Future studies should aim for more balanced sex representation to explore this aspect in greater detail. Although, this study employed a prospective design, which limits the ability to infer causal relationships between fasting and mood changes. Longitudinal studies or designs involving repeated measurements over time are recommended in future research to better assess long-term psychological effects of Ramadan intermittent fasting. Moreover, the number of fasting days was not recorded, and it is likely that female participants fasted fewer days due to menstruation. This represents a notable confounding factor, as variations in fasting exposure between male and female participants may have influenced mood outcomes—particularly in fatigue and tension subscales. Since this variable was neither measured nor controlled for, it limits the internal validity of sex-based comparisons and introduces uncertainty into the interpretation of mood changes. The sex imbalance and the overrepresentation of 4th-year students may limit the generalizability of our results. Future studies should aim for more diverse and balanced samples. As a self-report instrument, the BRUMS scale is subject to individual interpretation, mood recall bias, and variability in emotional self-awareness, which may influence the accuracy and consistency of reported mood states. While BRUMS is a validated and widely used tool, these inherent limitations should be considered when interpreting the results. Participants were not screened for psychiatric disorders as part of the study. The presence of undiagnosed or unreported mental health conditions may have influenced the mood scores and should be considered a limitation.

## Conclusion

This study presents a comprehensive analysis of the impact of RIF on various mood dimensions using the Brunel Mood Scale, shedding light on how demographic variables moderate these effects. Our findings support the hypothesis that RIF is associated with significant fluctuations in various psychological outcomes. Specifically, fatigue and tension increased during fasting, while confusion and depression decreased post-fasting, suggesting both physiological strain and emotional adaptation. Tension emerged as the most significantly affected domain, with notable associations across academic year and smoking status. These results indicate that RIF can meaningfully influence mood and anxiety-related symptoms, and these effects may vary based on sociodemographic factors. The findings highlight the need for targeted mental health support strategies for students during Ramadan, especially for those in higher academic years or with predisposing lifestyle factors.

## Data Availability

The raw data supporting the conclusions of this article will be made available by the authors, without undue reservation.
